# Supplementary material for: Are carbon emissions trading and green financial instruments synergistic? -Comprehensive quantitative research based on content analysis
Source: PLoS One. 2024 Mar 7;19(3):e0298601. doi: 10.1371/journal.pone.0298601 (PMC10919601; doi:10.1371/journal.pone.0298601)
Supplement: S1 Data — (ZIP) [file pone.0298601.s001.zip › Data/地级市Z_F工作报告.zip!/░▓╗╒/╦▐╓▌╩╨/└┤╫╘╣1⁄2╓┌║┼í╛┬φ┐╦╩2╛▌═°í┐_2016.docx]

2016年1月20日在宿州市第四届人民代表大会第五次会议上

市长 史 翔

各位代表：

现在，我代表市人民政府，向大会报告政府工作，请予审议，并请市政协委员和其他列席人员提出意见。

一、2015年及“十二五”工作回顾

过去一年，全市人民在省委、省政府和市委的坚强领导下，认真贯彻落实党的十八大、十八届三中、四中、五中全会和习近平总书记系列重要讲话精神，主动适应经济发展新常态，锁定“调转促”工作主线，迎难而上，主动作为，较好完成了市四届人大四次会议确定的主要目标任务。

初步核算，全市生产总值1235亿元，增长9%左右。财政收入125.6亿元，增长9.8%。社会消费品零售总额424亿元，增长12.3%以上。城镇、农村常住居民人均可支配收入23740元和9215元，分别增长8.2%、10.5%。节能减排达到省控目标，PM10平均浓度下降12.4%。动态指标继续保持全省前列，全市经济稳中有进、稳中向好，社会大局和谐稳定。

一是工业经济不断壮大。全市规模以上工业增加值380亿元，增长8.5%。规模以上企业达到1169户。全力推进“3111”工程，鞋服、板材、食品、建材、云计算、生化医药等首位产业逐步成长，实现产值480亿元，增长25%；首位产业税收10.5亿元，增长12%。战略性新兴产业产值首次突破百亿元，增长25%以上，为历史之最。园区活力不断增强，市经开区首位产业规模以上企业20家，宿马园区鸿丰生物等10个产值亿元以上项目投产，高新区云计算产业落户企业达92家，鞋城成为全国11个特色制鞋产业集聚区之一。

二是有效投入稳步增长。全年固定资产投资突破千亿元，增长20%，其中工业投资628亿元，增长18%。钱营孜低热值煤发电、科立华化工、林洋电子等450个亿元以上项目建设加快，吉美日用品、海升果胶、秉拓电子等90个亿元以上项目投产，安特食品等退城进园项目建成投产。德上高速砀山段、宿灵泗快速通道建成通车，郑徐客运专线、京沪高铁宿州东站改造及配套工程、宿淮铁路蒿沟站、宿萧砀快速通道和206国道改线工程建设加快，国省干线公路完成投资50.8亿元。积极对接省“三企一资”等重大招商引资活动，实际到位内资910亿元，增长25%；利用外资6.7亿美元，增长14%。

三是县域经济持续发力。县区主要经济指标增速连续两年高于全市平均水平，县域经济总量超千亿元，占全市的84%。四县一区财政收入增速高于全市2.5个百分点，对全市贡献率达73.7%。这充分说明，主攻县域经济，补上这个最大的短板，不仅完全正确，而且成效明显。

四是城乡建设突飞猛进。获批开展宿州市城市总体规划修编工作，四个县均完成总体规划修编。全市投资183亿元，实施重点工程176项，基础设施建设实现重大进展，产城一体步伐加快，旧城改造有序推进，市容市貌焕然一新。国家园林城市正式公示，新汴河景观带获批省级水利风景区，植物园一期建成开放。区域交通条件大为改善，宿城大外环即将全线贯通，环内面积超200平方公里，一个宜居宜业的新宿州已经展现在皖北大地。征迁机制日臻成熟，控违成效显著。扎实推进“555”工程，四个县建设重点工程80个，投资102亿元。第二批37个美好乡村示范村建设完成，第三批40个示范村建设稳步推进。5座垃圾焚烧发电厂和4座秸秆发电厂建设加快。农村电网改造升级全面铺开。

五是现代农业扎实推进。粮食生产“十二连丰”。现代农业“两区”建设加快推进，20个示范基地及园区投资13亿元，实现产值108亿元。加快培育新型经营主体，家庭农场、现代农业产业化联合体分别达到3641个和175个，农民专业合作社数量居全省首位。农产品加工产值920亿元，市级以上龙头企业发展到516家，萧县循环产业园成为国家农业产业化示范基地。砀山县列入全国电子商务进农村综合试点。连续十七年实现耕地占补平衡。淮水北调等重点水利工程加快推进。埇桥区荣获省“江淮杯”三连冠。新解决60万人饮水安全问题。新造林12万亩。大力实施扶贫开发“五个一”重点工程，359个贫困村实现包保全覆盖，投入扶贫资金3亿元，减贫11万人。

六是改革开放活力增强。大力简政放权，全市审批权力事项精减56.6%，涉企收费项目减少34%。全面彻底放开城镇落户限制，户籍制度改革蹄疾步稳。深化商事制度改革，新登记注册企业8890户，居全省前列。公务用车制度改革全面完成。文化体制改革、医药卫生体制综合改革持续深化。不动产统一登记改革顺利推进。实施金融“五个一”工程，贷款突破800亿元，新增存贷比70%，直接融资25亿元。设立“徽银”产业基金和“城镇化1号”基金，总规模30亿元。皖北制药、绿洲森工在主板上市辅导备案，2家企业在新三板挂牌，33家企业在省股交中心挂牌。进出口总额增长12.3%。

七是民生工程又好又快。全年民生支出249.4亿元，占财政总支出的84.7%。33项民生工程年度任务圆满完成。10件惠民实事顺利实施，新增纯电动公交车60辆，新投放公共自行车2500辆，完成中山街商品市场基础设施改造。新建各类保障房3.38万套，基本建成1.84万套，开工率、基本建成率居全省前列。城镇新增就业6万人，登记失业率控制在4.5%以内。“五险”参保180万人次，新农合参合率100%，城乡居民基础养老金标准提高到75元，发放城乡低保资金5.5亿元。

八是社会事业协调并进。新建、改扩建乡镇公办幼儿园125所，完成149所学校标准化建设，宿城一中城南学校建成使用。公共文化场馆免费开放，市文化馆通过国家一级馆评估。《宿州市志》出版发行。国内旅游收入增速居全省前列，砀山被评为“中国最美生态宜居旅游名县”，灵璧A级景区数量全省领先。卫生和计生工作扎实推进，泗县基层机构整合模式获国家推广。成功举办市第五届运动会。积极推行网格化新型社区管理模式，安全生产形势总体平稳，“治超”工作全省领先，应急管理体系不断完善，食品药品监管规范有序，信访秩序继续好转，“平安宿州”建设扎实推进。成功组织战时国防动员演练，双拥共建、人民防空工作成绩显著。荣获全国红十字会系统先进集体、全国防震减灾工作综合考核先进单位称号。民族宗教、外事侨务、物价、对台、档案、科技、气象、妇女儿童、残疾人等事业得到进一步加强。

在统筹做好各项工作的同时，我们始终高度重视政府自身建设。坚持“依法依规、集体决策、公开透明、终身负责”，推进法治政府建设，落实重大事项合法性前置审查制度，全面推行政府法律顾问制度，依法行政能力不断加强。扎实开展“三严三实”专题教育，严格执行“八项规定”，持续整治“四风”。深入推进“点燃激情、提升效能”活动。政务服务信息化平台建成运行。自觉接受人大监督、政协民主监督和社会舆论监督，办理人大代表议案和建议112件，政协建议案和委员提案266件。强化行政监察，推进审计监督、制度规范全覆盖，廉政建设弛而不息。

各位代表！

过去一年工作任务的顺利完成，标志着“十二五”发展的圆满收官。五年来，我们积极应对复杂多变的宏观经济形势，砥砺奋进、勠力同心，艰难困苦、玉汝于成，经济社会发展取得了一系列巨大的成就。

——这五年，是我市经济社会加速发展、综合实力显著提升的五年。生产总值连跨六个百亿元台阶，总量翻番，年均增长11.1%。财政收入总量翻了一番半，年均增长23.8%。固定资产投资由406亿元增加到1135亿元，年均增长25.7%。县区、园区发展全面提速，多点多极支撑的发展格局开始形成。在省政府目标管理绩效考核中，前四年，我市相继获得全省第六、第九、第八的成绩，2014年更是实现历史性突破，位列全省第五。可以说，过去五年，我们奋力谱写了浓墨重彩的发展篇章，经济增长自建市以来首次全面高于全省平均水平。今天的宿州，已经有了新的形象，站在了新的历史起点上。

——这五年，是我市经济结构加速调整、发展质量显著提升的五年。三次产业结构由27.9:37.9:34.2调整到22.1:41.4:36.5。财政收入占GDP比重由6.6%增加到10.2%。首位产业持续发力，规模以上工业企业数、总产值、亿元以上工业企业数分别是“十一五”的1.3倍、2.3倍、3倍，工业对经济贡献率大幅跃升。现代农业“两区”建设亮点纷呈，农业产业化综合水平稳居全省前列。云计算、大数据、生物医药等战略性新兴产业从无到有，快速崛起。现代服务业继续保持全省先进位次，社会消费品零售总额连续五年实现两位数增长。过去五年，我们着力扩大增量、优化存量、做大总量，为“调转促”奠定了坚实的基础。

——这五年，是我市基础设施加速建设、城乡面貌显著提升的五年。率先启动新型城镇化试点，相继开展城乡建设突破年、奋进年、提升年活动，累计投入资金802亿元，一批标志性工程相继建成。主城区实现“双75”目标，县城特色不断彰显，113个美好乡村示范村面貌焕然一新，“三线三边”环境综合治理成效显著。综合交通条件发生质的飞跃，宿州区位优势进一步凸显，在全省率先进入高铁时代，形成“两纵三横”铁路网；实现县县通高速，拥有“三纵两横”高速公路网；新增城市道路257公里，“六纵十横”路网格局形成，一级公路和农村公路互联互通达到新水平。以文化彰显城市魅力，连续举办奇石文化节、马戏节、大运河申遗等活动，城市凝聚力不断加强。过去五年，我们共同见证宿州变美变靓，大美宿州新画卷正徐徐展开，城市知名度和美誉度得到全面提升。

——这五年，是我市改革开放加速推进、动力活力显著提升的五年。顺利完成市、县（区）政府职能转变和机构改革。大力推进行政审批制度改革。开展金融创新，新增贷款是“十一五”的近3倍，存贷比提高10个百分点以上，金融机构增至16家。市城投集团等融资平台实现转型发展，完成市担保公司市场化改革、5家农信社改制。民营经济发展迅速，民营企业数量是“十一五”末的4倍，从业人员占全社会的96%，税收贡献率达86%。现代农业产业化联合体培育走在全国前列，土地承包经营权确权登记颁证工作走在全省前列，流转土地348万亩。宿州海关获批，出入境检验检疫局挂牌开检。累计利用外资、进出口总额，均是“十一五”的6倍。过去五年，我们打出一系列改革开放的“组合拳”，实现了第一波的“弯道超越”。

——这五年，是我市民计民生加速改善、社会事业显著提升的五年。城镇、农村常住居民人均可支配收入年均分别增长10.1%、14.1%，高于全省平均水平。58.2万农村贫困人口实现脱贫。连续五年实施33项民生工程和10件惠民实事。建成保障性安居工程637万平方米。社会保障体系更加健全，城乡基本养老、基本医疗保险和大病保险实现全覆盖。新增城镇就业16.3万人。过去五年，我们顺应群众过上更好生活新期待，大力推进基本公共服务均等化，人民群众幸福感、获得感、归属感明显增强。

——这五年，是我市城乡居民精神面貌加速转变、文明素质显著提升的五年。精神文明创建蓬勃开展，首次跻身省级文明城市，荣获全国双拥模范城等多个国家级荣誉称号。“中国好人”上榜16名，“安徽好人”44名，彭伟平获全国道德模范荣誉称号，“宿州好人”品牌享誉江淮大地，为全省之最。重拳整治非法营运三轮车、四轮车，交通秩序不断优化，扎实推进“四清”、“五化”，城市形象日新月异。不断加强市民素质和习惯的培养，讲文明、有礼貌、重秩序正逐渐成为宿州人的新特质。过去五年，我们持续培育践行社会主义核心价值观，不断弘扬时代的新风貌，传统文化的精髓在这片古老大地上结出了丰硕之果。

成绩令人振奋，经验弥足珍贵。我们深切体会到，必须始终坚持高举中国特色社会主义伟大旗帜，以习近平总书记系列重要讲话精神为引领，顺应大势、把握大局；必须始终坚持整体承接、双轮驱动、三区共进、四化同步，努力走好这一符合中央精神、省里要求、市情实际的发展路径，持之以恒抓主抓重、苦干实干、提速提效、争先争优；必须始终坚持发展是硬道理，全力抓好“调转促”，矢志不渝振兴县域经济，持续唱响工业“3111”和城乡建设“555”主旋律；必须始终坚持作风建设，持续“点燃激情、提升效能”，不断巩固同频共振、同向同行、共谋发展的良好局面；必须始终坚持民生优先，尊重人民意愿，促进公平正义，寻求最大公约数，汇聚发展正能量。所有这些，既是过去五年积累的宝贵经验，又是我们今后必须长期坚持的重要遵循。

在肯定成绩的同时，我们也清醒地认识到，全市经济社会发展中还存在不少困难和问题。主要是：经济总量小、均量低、发展不优不强的状况还没有根本改变，在区域竞争格局中压力很大；“调转促”任务艰巨，四化同步不够协调，县域经济不强，工业基础薄弱，战略性新兴产业支撑不足，现代服务业发展相对滞后；环境、土地、资金、劳动力等要素制约趋紧，实体经济发展步履维艰；城镇化水平较低，中心城区辐射带动能力弱，对工业化发展承载能力有限，城乡之间、县区之间发展不均衡；脱贫攻坚任务艰巨，社会保障、公共服务水平与群众期盼还有不小差距；政府职能转变还不到位，发展环境有待进一步优化，少数干部激情不足、为官不为、缺乏担当精神，“四风”、“小四风”等顽疾仍时有发生。对此，我们一定要保持谦卑，枕戈待旦，不负人民重托。

各位代表！

五年的开拓奋进，开启了实干兴宿的崭新局面。这是市委总揽全局、坚强领导的结果，是市人大、市政协、社会各界认真监督和大力支持的结果，更是全市上下同心协力、顽强拼搏的结果。在此，我代表市人民政府，向奋战在各行各业的全体劳动者，向离退休老同志，向各民主党派、工商联和人民团体，向中央和省驻宿单位、人民解放军驻宿部队、武警官兵、公安干警，向参与宿州建设的海内外企业家和客商、广大援宿干部，以及所有支持宿州发展的各界人士，表示衷心的感谢！

二、“十三五”发展总体思路和目标任务

“十三五”时期是全面建成小康社会的决胜阶段。综合分析，与各地一样，我市发展仍处在可以大有作为的重要战略机遇期。我国经济长期向好的基本面没有改变，“一带一路”、长江经济带、中原经济区等战略深入实施，供给侧改革和新型城镇化的推进，将持续释放巨大需求；我省率先系统推进全面创新改革试验，皖北振兴、南北对接深入推进，多重支持政策叠加；我市通过多年努力，发展优势厚植，区域、资源和市场优势进一步凸显，长期有效投入效应不断释放，工业化城镇化向更高水平跃升，“狠抓落实、实干兴宿”的氛围更加浓厚，这都为“十三五”发展汇聚了磅礴力量。同时必须看到，影响经济发展的不稳定、不确定因素依然突出，如期全面建成小康社会既具有充分条件，也面临艰巨任务。我们要准确研判，积极应对，认识新常态、适应新常态、引领新常态，把宿州发展推向新的更高水平。

根据党的十八届五中全会、省委九届十四次全会精神和市委四届八次全会通过的《关于制定国民经济和社会发展第十三个五年规划的建议》，我市“十三五”发展的指导思想是：全面贯彻党的十八大和十八届三中、四中、五中全会精神，深入贯彻习近平总书记系列重要讲话精神，认真落实“五位一体”总体布局和“四个全面”战略布局，牢固树立创新、协调、绿色、开放、共享的发展理念，坚持发展第一要务，以做大总量、提质增效为中心，以调结构转方式促升级为引擎，以增进人民福祉为目的，加快形成引领经济发展新常态的体制机制和发展方式，确保如期全面建成小康社会，奋力开创创新型“四个宿州”建设新局面。

在此框架下，市政府编制了“十三五”规划《纲要（草案）》。综合考虑，今后五年经济社会发展的主要目标是：按照“皖北领先、省内争先”的总体要求，着力打造“一区两中心五基地”。经济保持中高速增长，主要经济指标聚焦“保八争六、争先进位”目标，加快推进总量、质量和均量全面提升，力争经济总量在全省进前六，财力水平进前八。

——经济实力明显提升。三次产业结构持续优化；生产总值超过2000亿元，年均增长9%左右；财政收入达到200亿元，年均增长10%左右；固定资产投资年均增长15%以上，主要经济指标增速位居全省前列。

——人民生活质量和水平普遍提高。城镇、农村常住居民人均可支配收入年均分别增长9%、10.5%，公共服务体系更加健全，社会更加和谐稳定。提前两年打赢脱贫攻坚战。

——城乡发展更加协调。城镇化水平和质量进一步提高，中心城市实现“双100”目标，四个县城进一步提质扩容，重点城镇能级迅速提升，美丽乡村建设深入推进。

——生态环境总体改善。主要污染物排放、万元GDP综合能耗达到省控目标，万元工业增加值用水量显著降低。城乡环境综合治理成效明显，生态质量明显改善。

——文明程度显著提高。中国梦和社会主义核心价值观更加深入人心，文化事业和产业更加繁荣，“16城同创”取得新突破。

——改革开放深入推进。重点领域和关键环节改革取得决定性成果，政府职能实现根本性转变。对内对外开放的广度和深度不断拓展，区域合作更加紧密。

为此，重点抓好以下五个方面：

（一）突出创新发展，推动经济实力和产业结构“双提升”。深入实施“4105”行动计划，抓好十大重点工程，努力实现中高速增长和中高端发展。持续推进工业强市兴县，加快做大首位产业，大力推进一、二、三产业融合发展，打造现代产业新体系。积极开展全面创新改革试验，加快形成有利于“调转促”的创新驱动发展新机制。构建创业创新载体平台，推动大众创业、万众创新。以经济体制改革为重点，打好改革攻坚战，激活发展新能量。加快国家智慧城市试点建设，拓展网络经济空间，培育一批“宿州云”知名品牌。

（二）突出协调发展，推动城镇建设和乡村治理“双提质”。坚持“一尊重、五统筹”，着力提高城市工作水平，加快构建“一核、多轴、两板块”的城镇空间结构。强化城乡基础设施建设。推进以人为核心的新型城镇化，落实户籍制度改革，促进农业转移人口与城镇居民享有同等权利。争取三年时间全面完成市县（区）连片棚户区改造任务。加快美丽乡村建设，深化“三线三边”环境综合治理。广泛开展群众性精神文明创建活动，争创全国文明城市。擦亮城市名片，着力打造孝文化名城、儿童幸福生活城、关爱弱势群体模范城。

（三）突出绿色发展，推动生产方式转变和生态环境改善“双提速”。坚持把生态文明建设融入经济社会发展全过程，促进形成城镇建设有序、产业定位清晰、人与自然和谐相处的区域空间开发总体战略格局，建设“天蓝、地绿、水净”的美丽宿州。编制市县主体功能区规划。节约集约利用资源，实行最严格的水资源管理制度、节约用地制度、耕地保护制度。推进节能降耗，大力发展循环经济。加大环境治理力度，加强生态文明制度建设，着力改善生态环境。

（四）突出开放发展，推动深化改革和合作交流“双提效”。融入国家“三大战略”，加强区域合作交流。积极参与淮海经济区核心区一体化建设，推动淮海城市群建设上升为国家战略。加快与徐州对接连通、融合发展，建设具有较强竞争力的宜居宜业中心城市。深化南北合作，推动县域结对共建园区。提升招商引资水平，大力发展外向型经济，打造皖北开放新高地。强化大通道、大平台、大通关建设，加快基础设施互联互通，实现与周边市县全面快捷的道路连通，加强口岸和特殊监管场所建设，推进建设内陆“无水港”。

（五）突出共享发展，推动居民生活水平和文明素质“双提高”。确保城乡居民收入增速快于经济增长，达到全省平均水平，居民生活质量普遍提高。现行标准下农村贫困人口提前两年实现脱贫，贫困村全部出列，贫困县区全部摘帽。千方百计稳岗增岗，促进就业创业。推动我市由“民工大市”向“技工大市”转变。推进健康宿州建设，积极申办2022年全省运动会。健全教育、文化、医疗、养老等公共服务体系，完善社会保障，兜住民生底线，实现全体居民共同迈入全面小康社会。

三、2016年的主要工作

今年是全面建成小康社会决胜阶段的开局之年，也是推进结构性改革、实施“调转促”行动计划的攻坚之年。综合考虑，经济社会发展主要预期目标是：全市生产总值增长9%左右，财政收入增长9%左右，固定资产投资增长15%左右，规模以上工业增加值增长9%左右，社会消费品零售总额增长11%左右，城镇、农村常住居民人均可支配收入分别增长8.5%、10.5%左右，主要动态指标继续位居全省前列。我们要适应经济发展新常态，以“4105”为主抓手，去产能、去库存、去杠杆、降成本、补短板，增强持续增长动力，努力实现“十三五”发展良好开局。

（一）实施“调转促”行动计划

狠抓招商引资。坚持以“工业项目、招大引强、税源企业和环境保护”为招商导向，实际到位内资增长15%，实际利用外资增长6%。制定首位产业链招商路线图，大力推进精准招商、产业链招商，在关键环节、核心项目上实现新突破。坚持全员招商与专业招商相结合，继续实施招商周制度，配齐配强各级园区招商队伍，加快建立各类专业招商小分队，集中精兵强将，主攻重大项目。完善招商引资考核办法，加大工业首位产业、战略性新兴产业、开竣工项目的考核权重。

做强传统产业。实施传统产业改造提升工程，围绕“互联网+”行动计划，通过新技术、新业态、新模式，加快技术改造和智能化改造，推动传统产业向中高端迈进。千方百计降低实体经济运营成本，在进一步正税清费、降低企业财务成本、制度性交易成本等方面打出“组合拳”。积极稳妥处置“僵尸企业”，倒逼过剩产能退出。大力发展民营经济，对所有新登记注册企业一路绿灯，免征企业开业前的一切行政性收费，让个体私营经济在宿州大地遍地开花。坚持经济工作项目化、项目工作责任化、责任追究制度化，全面落实“四督四保”要求，加快国能煤制气等项目进度，力促砀山海升果酒工业园等项目开工，确保左右家私等项目建成投产，完成雪龙纤维等退城进园项目建设任务，全年工业投资完成730亿元，实现规模以上工业增加值420亿元，新增规模以上企业80户。建立“大新专”、“调转促”项目库，重点项目储备规模超万亿元。

壮大战略性新兴产业。加快申报和建设云计算产业园、上海张江萧县高科技园等省级战略性新兴产业集聚基地，打造经济发展新的增长极；着力培育生物医药产业，加快推进亿帆药业、一灵药业等项目建设；着力培育高端装备制造产业，加快推进江淮重工、龙华矿机等项目建设；着力培育新能源产业，加快推进坤盛新能源等项目建设；着力培育节能环保产业，加快推进泗县室内车内环保产业基地等项目建设；着力培育新材料产业，加快推进碳纤维、石墨烯、增材材料研发制造。实施创新驱动发展工程，深入推进大众创业、万众创新，加快培育一批“专精特新”中小企业。力争战略性新兴产业产值增长30%以上。

提升现代服务业。高起点编制现代服务业发展总体规划。大力发展商贸物流业，开工建设苏宁云商项目，加快国购广场等项目建设。发展壮大文化旅游产业，推进南翔文化商业街、孝文化产业园建设，抓好皇藏峪5A级景区创建，大力发展蔡洼红色旅游，争创4A级景区2家。推进电子商务产业集聚园区建设，加强与国内知名电商合作，加快阿里巴巴宿州产业带运营，打造中国网库“宿州电商谷”，推进“千家电商进园区”活动，大力发展农村电子商务。实现服务业增加值增长10%左右，新增2家省级服务业集聚区。

强化金融支撑能力。继续实施金融“五个一”工程，加强银企对接，扩大信贷投放，全年新增贷款130亿元，存贷比达到55%。积极引进中信银行等在我市设立分支机构，鼓励优质农商行异地设立分支机构。加快企业上市步伐，力争主板上市1家、辅导备案2家，新三板挂牌5家，省股交中心挂牌10家。利用多种融资工具，实现直接融资60亿元。认真落实“4321”政银担风险分担合作机制，提高政策性融资担保机构担保能力，加大“税融通”推广力度。创新金融产品服务，规范发展互联网金融。加强信用体系建设，防范化解金融风险，持续优化金融生态环境。

（二）推进县域经济上台阶

做大首位产业。继续强化工业强县的理念，深入持久地实施“3111”工程，完成三个“10”的项目建设任务，实现县区首位产业产值、投资、税收增速均高于全市工业发展水平10个百分点以上，夯实县域发展基础。统筹首位产业布局，鼓励埇桥发展纺织服装、家具板材产业；萧县发展建材、化工产业；砀山发展果蔬食品产业；灵璧发展机械电子、食品加工产业；泗县发展机械电子、品牌服装产业。推进乡镇工业项目向县区开发区集中，鼓励有条件的乡镇因地制宜发展特色产业。

加大扶持引导。按照“一县一策”的原则，制定完善主导产业实施路线图、招商目标企业导引图和项目库，打造产业链的“升级版”。认真落实金融支持县域经济发展的实施意见、金融工作考核办法，持续增强融资担保功能，落实商业银行信贷投放承诺制度，新增存款主要用于当地。继续按照以项目定用地、以投资额定用地的原则，合理配置土地指标，保障发展用地需求。建立完善权责对等的行政管理体制，赋予县区更大的自主权。

强化考核调度。完善县域经济发展考核体系，不断加大首位产业产值、工业税收、重点项目建设等指标权重，奖优罚劣，鼓励县区争先进位、勇创一流。继续坚持经济发展定期调度、首位产业培育专项调度，促进县域经济水平整体上台阶。

（三）提升城市工作水平

大力推进新型城镇化。继续实施新型城镇化试点三年行动计划，加快提高户籍人口城镇化率，促进以人为核心的城镇化。扎实推进户籍制度改革，全面落实农业转移人口“五权不变”、“五有并轨”，有序推进农业转移人口市民化。

加快城乡建设。加强城市布局谋划、功能规划、空间管控规划和城市设计工作，充分发挥规划的引领作用。统筹各类空间性规划，主动推进“多规合一”。投资100亿元，实施四大类105项重点工程。主城区力争实现“双80”目标。完善汴河新区、高铁片区、城南片区、城东片区公共服务设施，建设汽车客运中心站及配套公交枢纽站，加快建设植物园、动物园。以人民路和拂晓大道两侧、道东片区等区域为重点，实施棚户区改造210万平方米。积极化解房地产库存，棚户区改造货币化安置比例不低于50%。坚持“三治三增三提升”，加强县、镇、村建设，继续实施“555”工程。县城5项工程：完成整体棚改任务的三分之一以上；建成一个海绵城市或地下管廊示范工程；打通一批断头路；开工建设一个亿元以上的PPP项目；新建绿道10公里。县城5项任务：完成PPP项目融资5亿元；完成补充耕地指标3000亩；完成征迁整理净地1000亩；编制完成县城海绵城市和地下管廊专项规划；编制完成县城及重点镇棚户区改造规划和计划。镇村5项工作：基本完成乡镇政府驻地建成区整治建设任务；建立完善农村生活垃圾收集、转运机制，实现农村生活垃圾治理全覆盖；完成美丽乡村示范村道路和污水管网建设；新增10个以上集体经济收入过10万元的村；完成农村集体土地确权工作。

完善基础设施。谋划推进徐淮宿阜城际铁路、合青高铁泗县段、徐宿阜高速公路、徐州地铁萧县延长线等项目，加快京沪高铁宿州东站改造及配套工程、宿淮铁路蒿沟站建设，确保郑徐客运专线正式通车。加快国省干线一级公路改造，实现宿萧砀快速通道、206国道改线工程全线通车，力争104国道泗县段改建工程开工。建设人民路北延至徐州双向八车道标准的符离大道，促进宿州与徐州全面融合发展。完善城市路网，新建206国道北段等项目，加快推进西外环等项目，完成老南外环路提升改造，提升二徐路、高速南出口等城市重要路段。提高农村公路延伸、畅通和养护管理水平。推进宿州机场、砀山通用机场项目，谋划主城区轨道交通建设。继续实施农村电网改造升级工程。建成西气东输宿州天然气综合利用工程。实施“宽带宿州”战略，建设“无线城市”，扩大无线网络覆盖范围。

提高管理水平。创新城市治理方式，推进城市精细化管理。加强垃圾处理设施建设，下决心解决“垃圾围城”问题。坚持“为了人而管好城市”，加强城市公共安全管理。加快智慧城市建设，拓展“城管服务超市”功能，推进城市综合管理智慧化。加强住宅小区物业管理，强化高层住宅二次供水监管。深入开展“16城同创”，践行社会主义核心价值观，加快完善市民公约、乡规民约、行为规范，推动“宿州规矩”成为“宿州习惯”。鼓励企业和市民参与城市建设管理，实现共治共管、共建共享。

（四）加快推进农业现代化

转变农业发展方式。实施农业现代化推进工程，以“两区”建设为平台，大力推进现代农业“两大行动计划”和“四大提升工程”，努力打造全国创新型农业现代化先行区。推动粮经饲统筹、农林牧渔结合、种养加一体、一二三产融合发展。加快培育新型农业经营主体，创建市级现代农业产业化联合体50个、示范家庭农场50家、示范合作社50个。用工业的理念发展农业，加大招商力度，狠抓现代农业园区和项目建设。实施农业产业化规模提升行动计划，实现农产品加工产值1100亿元，市级以上龙头企业超600家。积极发展特色农业、休闲观光农业和农产品精深加工业，提高农业综合效益和竞争力。因地制宜推动适度规模经营，支持农民合作社拓宽服务领域，支持农民以土地经营权入股合作社、龙头企业。培育新型职业农民1300人。

发展高效农业。开展粮食绿色增产增效模式攻关，确保粮食安全。加强农田水利等基础设施建设，建设高标准农田。加大农业科技创新，提高农业装备和技术水平。加强社会化服务体系建设，推广农业物联网等新技术，推进“信息进村入户工程”。加强农产品流通设施和市场建设，强化农产品质量安全监管，积极发展品牌农产品，让群众吃上放心粮油、果蔬。

推进美丽乡村建设。坚持发展中心村、保护特色村、整治空心村，不断完善基础设施和公共服务体系，再建40个美丽乡村示范村，加快实现“以点带面”的战略转换，支持泗县整县推进试点工作。推进“三线三边”环境综合治理，优化农村居住环境。

（五）扩大改革开放步伐

推进重点改革。着力加强供给侧结构性改革，加快推进各项国家级和省级改革试点任务。稳妥推进农村土地制度改革，完善三级土地流转服务体系，加快建立统一规范的农村产权交易平台，积极推进农村承包土地经营权抵押贷款试点。统筹推进国资国企、财税金融、医药卫生、供销社、司法、文化等体制改革，加快形成系统完备、科学规范、运行高效的体制机制。

夯实园区平台。启动实施园区转型升级工程，完善园区的水、电、气、路等配套功能，全面提高园区土地利用率，严把项目投资强度、容积率、能耗等准入关，着力提升园区专业化发展水平。市经开区重点发展生化医药产业。宿马园区重点发展食品（农产品）深加工、专用车及机械装备制造产业。高新区重点发展云计算和智能制造产业。鞋城加快实施“建链、补链、强链”工程。深化泗涂产业园、萧县泉山工业园等园区合作共建。

扩大对外开放。拓宽对外开放领域，全面加强区域合作交流。着力推动开放型经济发展，大力培养外向型企业，优化进出口结构。引导有实力的企业采取贸易、投资、工程承包等方式开展对外合作。实现宿州海关正式开关。推广上海自贸区可复制改革试点经验，进一步优化通关、结汇、退税等进出口环境，保持全市进出口总额增速位于全省第一方阵。

（六）加大脱贫攻坚力度

增强贫困地区发展能力。坚持精准扶贫、精准脱贫，全面落实“单位包村、干部包户”工作机制，实现359个贫困村派驻“脱贫攻坚第一书记”全覆盖，完成90个贫困村、13.8万贫困人口脱贫任务。深入推进扶贫开发“4+3”工作，建档立卡摸清“扶持谁”，各尽其责明确“谁来扶”，精准施策找准“怎么扶”，规范有序解决“如何退”。大力推进产业扶贫，实施“一村一品”产业推进行动，在1.1万个贫困户开展光伏扶贫，建设村级光伏电站65个，积极创建国家电商扶贫试点。实行就业扶贫，促进6万有劳动能力的贫困人口就业。易地搬迁贫困人口7000户。

促进贫困地区民生改善。实施县乡公路和乡村道路畅通工程，推进贫困村饮水安全巩固提升、电网改造升级、宽带网络覆盖，完成贫困地区农村危房改造1万户。免除贫困家庭高中学生学杂费。加大因病致贫群众医疗救助力度，把完全或部分丧失劳动能力的贫困人口全部纳入农村低保，并逐步提高标准。

（七）维护社会和谐稳定

统筹社会事业。合理配置、优化布局教育资源，加强教师队伍建设，大力改善薄弱学校基本办学条件，促进教育公平。继续实施学前教育三年行动计划，完成155所义务教育学校标准化建设，加快普及高中阶段教育，加快职教园区建设，支持高等职业教育和高校发展。完善公共文化服务体系，促进文化服务设施规范化，提升市博物馆、文化艺术中心开放工作。加强文艺精品创作，打造人文荟萃、特色彰显的宿州文化。做好大运河申遗后的保护利用工作。推进市立医院新区、市妇幼保健院等项目建设，提升县、乡、村医疗服务能力。实施好全面两孩政策，促进人口均衡发展。推动文化、体育场地向社会开放。实施质量品牌提升工程，开展“质量强市提升年”活动。扎实推进第三次全国农业普查。启动“七五”普法规划，加强国防动员、人防和民兵预备役建设，争创全国双拥模范城。加强民族宗教、外事侨务、防震减灾、残疾人、红十字、档案、对台、气象等工作。

创新社会治理。健全安全生产责任体系，加强道路交通、煤矿和非煤矿山、危险化学品、消防等重点行业领域安全监管，加大科技“治超”力度，严防重特大事故发生。强化食品药品安全监管，建成市级检验检测中心。推行法治信访、网上信访，集中化解信访积案。完善预警应急机制，提升突发事件防范、应对和处置水平。全面推进城镇“四有”社区和农村社区建设试点工作。严密互联网管理。完善立体化、数字化治安防控体系，依法严厉打击各类违法犯罪活动。

优化生态环境。坚守耕地保护、开发强度、生态保护“三条红线”，深入实施大气、水、土壤污染防治行动计划，加强环境空气质量监测能力建设。下决心加快园区污水处理厂和管网建设，确保工业污染源全面达标排放。加强饮水水源地保护，实施地下水超采区综合治理，再解决71万人饮水安全问题。加强重点河流流域环境治理，加快黄河故道生态综合开发，继续实施清水工程，积极申报新汴河城区段为国家级水利风景区。开展矿山地质环境恢复和综合治理，继续推进采煤沉陷区综合治理。持续实施森林增长工程。加大农业面源污染防治力度，加强秸秆综合利用和禁烧工作。

（八）切实保障改善民生

完善就业和社会保障体系。加大公共就业创业服务体系建设，促进高校毕业生、农村富余劳动力等重点人群就业，城镇新增就业3.5万人，城镇登记失业率控制在4.5%以内。实施人才高地建设工程，加大创新创业人才引进力度，与省内外高校开展人才培养与交流工作。实施全民参保计划，加大社会保险扩面征缴力度，落实社会保险关系转移接续政策，“五险”参保185万人次。开展应对人口老龄化行动，加快发展养老服务业，全面实施机关事业单位养老保险制度。启用低保信息系统，完善医疗救助“一站式”服务。高度重视农村留守儿童、妇女、老年人关爱帮扶体系建设，做好生活无着人员社会救助工作，保障孤儿基本生活。这是各级政府的重要责任！

实施民生工程和惠民实事。在深入推进33项民生工程的基础上，再办10件惠民实事，让群众更多分享改革发展成果。

（1）建设一批市民农园。

（2）新建改建一批公交停保场，新增一批纯电动公交车，新增和调整一批公交线路，建设公交智能调度系统。

（3）在人口密集区新建一批足球场、篮球场和健身路径带。

（4）启动“公厕第五空间”工程，新建和改造提升一批公厕，完成对市区所有旱厕的改造。

（5）新建改建100座乡镇公厕。

（6）改造市、县城区老旧电线。

（7）关闭非法自备井和城市自来水管网覆盖区内的自备井。

（8）继续增加停车位，规划建设一批停车场。

（9）建设一批供热示范区。

（10）改造消防给水管网，新增和维修市政消火栓。

四、进一步加强政府自身建设

推动新常态下的新发展，要求各级政府必须不断加强自身建设。我们要十分珍惜全市上下宝贵的发展势头，勇于担当实干兴宿的历史使命，朝夕勤勉，永不懈怠。

依法行政，建设法治政府。贯彻落实《法治政府建设实施纲要》，努力建设职能科学、权责法定、执法严明、公开公正、廉洁高效、守法诚信的法治政府。坚持“依法依规、集体决策、公开透明、终身负责”，切实提高决策质量。加强政府立法工作，执行政府法律顾问制度，所有政府决策必须于法有据。深化行政执法体制改革，健全行政裁量权基准制度，全面落实行政执法责任制。加大政务公开，及时回应社会关切。自觉接受人大法律监督、政协民主监督，切实办好人大代表议案和建议、政协建议案和委员提案，广泛听取人民团体意见，全面接受新闻媒体和人民群众监督。

服务至上，建设为民政府。保持公仆情怀，强化为民之责，以“三严三实”为行为准则，改进工作作风，密切联系群众，持续巩固教育实践活动成果。创新群众工作方法，加强民意调查、网络问政、调研问需，完善群众诉求反应反馈机制，打通联系服务群众“最后一公里”。推广“互联网+政务服务”，完善政务服务信息化平台建设，确保行政审批、便民服务项目全覆盖，实现网上办成事。

简政放权，建设高效政府。推进政府职能向提供公共服务、营造公平市场环境转变，更好地服务民生、推动发展。加快建设标准统一、资源共享的清单运行平台，增强放权的协同性、监管的有效性、服务的便利性。深化行政审批制度改革，持续推进“放、管、服”，依法下放权力、简化环节、优化程序，大力推行并联审批，积极打造最少环节、最短时限、最低收费、最优服务的一流政务环境。

狠抓落实，建设担当政府。与时俱进加强学习，努力成为新常态下各项工作的行家里手。直面供给侧、结构性、体制性矛盾，战略上坚持持久战，战术上打好歼灭战，稳扎稳打解决问题，真正做到守土有责、守土负责、守土尽责。健全督查激励问责机制，强化调度、定期问效，严格考核、奖优罚劣，重拳治理懒政、怠政，坚决纠正不作为、慢作为、乱作为。

永葆清正，建设廉洁政府。厉行勤俭节约，严控“三公经费”。进一步落实“八项规定”和省市“三十条”要求，严防“四风”反弹。贯彻《廉洁自律准则》和《纪律处分条例》，构建“不想腐”的自律机制。用制度管权管事管人，推动审计监督和制度规范全覆盖，完善“不能腐”的制约机制。强化“不敢腐”的惩戒机制，以零容忍的态度惩治腐败，让顶风违纪者得不偿失，让心存侥幸者望而生畏。

各位代表！

我们告别了辛勤耕耘、硕果累累的“十二五”，迎来了充满希望、大有可为的“十三五”。雄关漫道真如铁，而今迈步从头越。让我们紧密团结在以习近平同志为总书记的党中央周围，在市委的坚强领导下，解放思想、开拓创新，埋头苦干、励精图治，为建设创新型“四个宿州”、全面建成小康社会而努力奋斗！
